# A case report of a distal radius fracture treated with a dorsal spanning plate augmented with fragment specific fixation

**DOI:** 10.1093/jscr/rjae260

**Published:** 2024-04-24

**Authors:** Andrew L Chen, Evan J Hernandez, Brendan J MacKay

**Affiliations:** Department of Orthopedic Surgery, Texas Tech University Health Sciences Center, Lubbock, TX 79430, United States; Department of Orthopedic Surgery, Texas Tech University Health Sciences Center, Lubbock, TX 79430, United States; Department of Orthopedic Surgery, Texas Tech University Health Sciences Center, Lubbock, TX 79430, United States

**Keywords:** distal radius fracture, comminuted, intra-articular, dorsal spanning plate, augmented fixation

## Abstract

Dorsal spanning plates are frequently utilized to manage comminuted intra-articular distal radius fractures, but there is little literature on combining them with augmented fixation in complex cases. We present a 43-year-old man who fell 5 ft onto his outstretched right hand. On examination, there was gross swelling and tenderness of the right wrist with no neurovascular deficit. Radiographs confirmed a comminuted intra-articular displaced distal radius fracture. He was treated with a dorsal spanning plate fixation combined with radiostyloid and volar buttress plates. Follow-up at 2 months showcased intact hardware with good fracture healing. The dorsal spanning plate was removed 4 months after the procedure with radiographs demonstrating adequate alignment and healing without failure. The patient reported no complaints and minimal functional disability. We highlight a case of augmenting a dorsal spanning plate with fragment-specific plate fixation for a comminuted intra-articular displaced distal radius fracture.

## Introduction

Distal radius fractures are one of the most commonly encountered fractures in the USA with an estimated frequency greater than 640 000 per year, representing 44% of all fractures [1, 2]. There are a variety of surgical management options available each with their own indications. The use of dorsal spanning plates is an effective technique for highly comminuted intra-articular distal radius fractures [3]. Although fragment-specific fixation exists where a multitude of plates are used to stabilize specific articular segments, there is little literature on combining dorsal spanning plates with these techniques [4]. Here, a 43-year-old patient who had his distal radius fracture treated with an augmented dorsal spanning plate fixation technique is described.

## Case presentation

**Figure 1:**
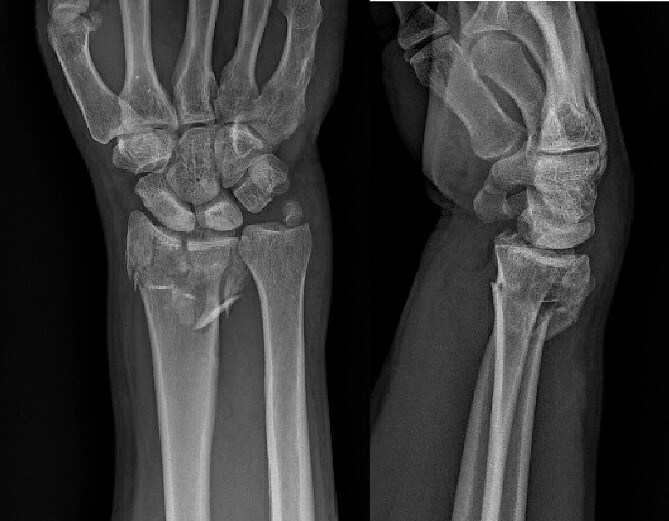
Preoperative radiological assessment of the patient with postero-anterior (PA) and lateral views of the right wrist.

**Figure 2:**
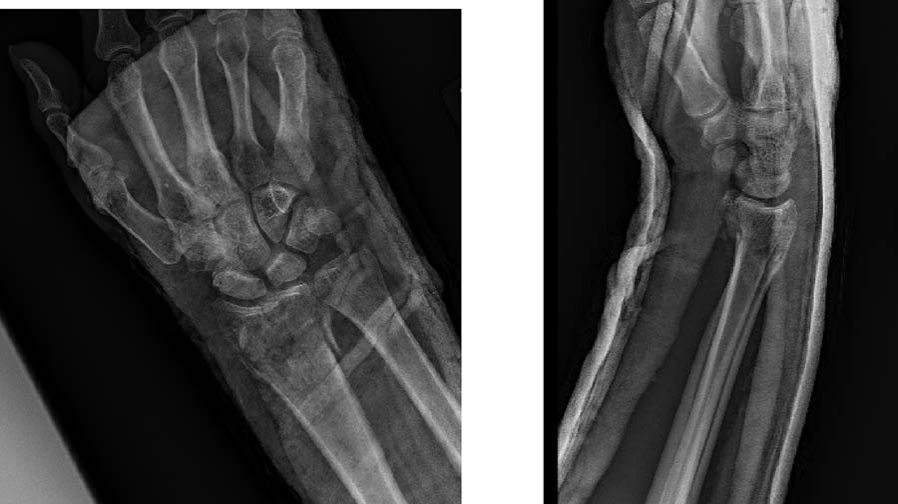
PA and lateral postsplint films.

A 43-year-old man presented to the emergency department after falling ~5 ft from a ladder onto his outstretched right hand. He reported immediate pain and inability to bear weight through his right wrist. On physical examination, there was right wrist swelling and limited range of motion (ROM). The skin was intact, and neurovascular exam was normal. The patient had no other injuries and no pertinent medical history. Initial radiographic imaging confirmed a comminuted intra-articular distal radius fracture with dorsal displacement along with an ulnar styloid fracture (Fig. 1). The patient was placed in a sugar-tong splint and followed-up in clinic a week later with surgical treatment planned (Fig. 2).

**Figure 3:**
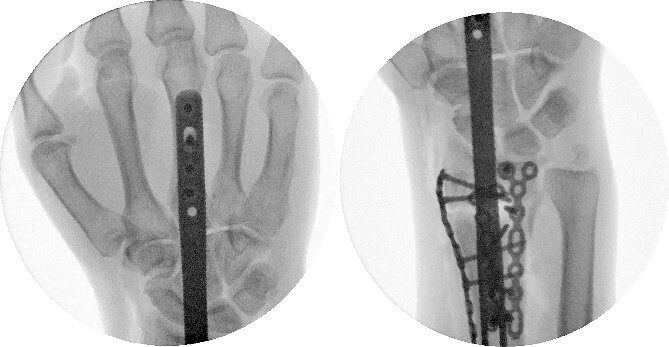
Intraoperative fluoroscopy with a PA view.

**Figure 4:**
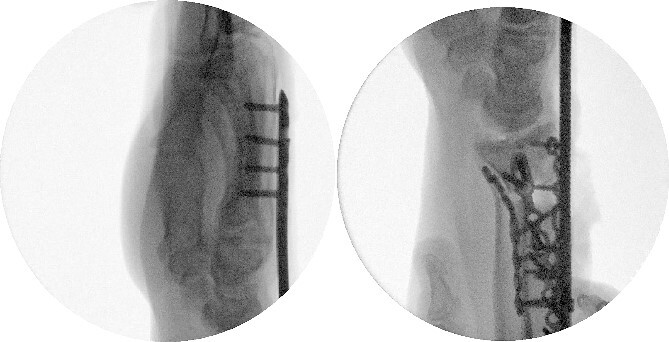
Intraoperative fluoroscopy with a lateral view.

A dorsal spanning plate was planned to treat the fracture. An incision was made over the third dorsal compartment followed by release of the fourth dorsal compartment to fully visualize the fracture. Once the plate was placed, intraoperative fluoroscopy revealed that that radial styloid was still displaced. After releasing the first dorsal compartment and brachioradialis, a radiostyloid plate was then placed. Additionally, the volar ulnar corner of the radius was still not reduced properly, so a Protean fragment specific volar buttress plate was placed. Final intraoperative fluoroscopy films revealed satisfactory reduction and alignment (Figs 3 and 4).

**Figure 5:**
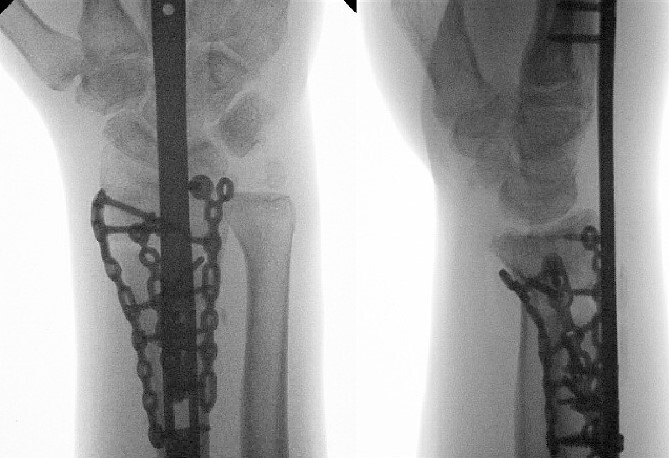
Radiological assessment at 2 months postprocedure with PA and lateral views.

**Figure 6:**
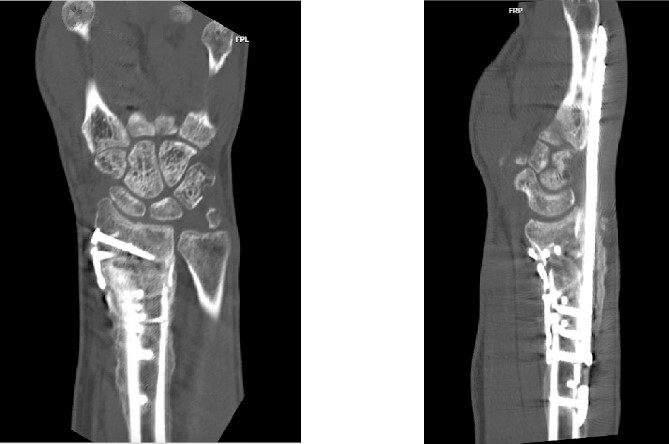
CT assessment at 10 weeks postprocedure with coronal and sagittal cuts.

**Figure 7:**
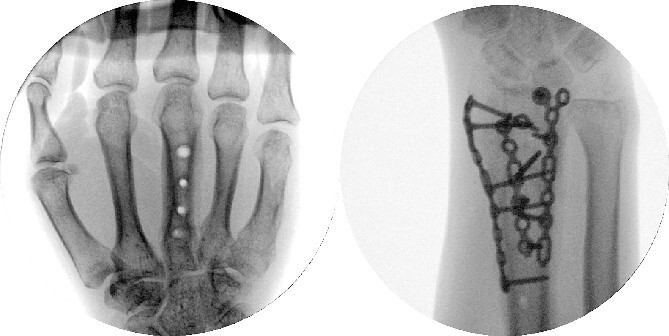
Dorsal spanning plate hardware removal at 4 months with a PA view.

Two weeks following the procedure, the patient returned to the clinic for a follow-up visit. The wrist was swollen and ROM was limited. Hand motor functions and neurovascular exam were fully intact. Radiographs taken in clinic showed intact hardware with healing fracture. At 2 months follow-up, radiographs showed intact hardware with interval bone bridging across fracture sites. Radiographic parameters including articular alignment, radial height, radial inclination, and volar tilt were in acceptable ranges (Fig. 5). The patient reported no concerns or complications. A computed tomography (CT) scan was obtained at 10 weeks after the procedure and confirmed adequate bony healing and alignment without failure (Fig. 6). The dorsal spanning plate was eventually removed 1 month later and 4 months since the initial operation (Fig. 7).

The patient followed-up in clinic 1 week after hardware removal. Clinically, the patient had no complaints. ROM was limited with both flexion/extension and supination/pronation. A QuickDASH assessment was obtained with a score of 25 out of 100, equating to minimal functional disability overall. Specifically, the patient reported moderate difficulty with washing his back and manual labor, and severe difficulty with impact recreational activities. The patient was to return to clinic in 4–6 weeks after undergoing physical therapy /occupational therapy to reassess ROM and functionality; unfortunately, the patient was lost to follow-up.

## Discussion

Distal radius fractures are one of the most common fractures encountered among adults in the USA. Depending on the type of fracture, different surgical techniques can be used including external fixation, volar locking plates, dorsal spanning plates, and fragment-specific fixation.

Dorsal spanning plates are commonly used for comminuted intra-articular distal radius fractures, but rarely are they combined with fragment specific fixation. In the literature review, there was only one study found that looked at augmentation of dorsal spanning plate fixation [5].

To our knowledge, there is no specific literature reported on combining dorsal spanning plate with radiostyloid and volar buttress plates for comminuted intra-articular distal radius fractures. Our case showcased its effectiveness in achieving acceptable radiological alignment in our patient. It should be noted that during the procedure, we opted to place the volar buttress plate through the dorsal incision to minimize soft tissue damage. After opening the first dorsal compartment to put down the radiostyloid plate, we released the pronator quadratus to access the displaced volar ulnar corner of the radius and reduced it with the buttress plate. This marks an intraoperative technique that could potentially be utilized in future cases.

Furthermore, even though retrieval of the dorsal spanning plate did not occur until 4 months after the procedure, our patient’s CT demonstrated adequate fracture consolidation, alignment, and readiness for hardware removal by the 10-week mark. Traditionally, dorsal spanning plates are left in place for at least 12 weeks to ensure adequate fracture consolidation [6, 7]. It is likely that additional augmentation of the dorsal spanning plate with selective open reduction internal fixation in our case may have decreased time to acceptable fracture healing for plate removal. As a result, this would theoretically result in earlier ROM and return to normal activities, potentially improving clinical outcomes. However, our case was limited in its ability to adequately assess final ROM and functional abilities due to loss to follow-up.

Longer term data with larger sample sizes and adequate follow-up are needed to further support the necessity and efficacy of augmenting dorsal spanning plate fixation for complex comminuted intra-articular distal radius fractures and analyze its ability to contribute to earlier hardware removal, rehabilitation, and return to function.

## Conclusion

Our case report showcases an uncommon instance of a comminuted intra-articular distal radius fracture requiring dorsal spanning plate in conjunction with fragment specific fixation. The combination of dorsal spanning plate with fragment specific fixation may provide better radiological alignment and earlier bony healing in complex distal radius fractures compared to dorsal spanning plate alone. Further prospective studies are required to assess the long-term radiological and clinical outcomes of this augmented technique.
